# Society News

**DOI:** 10.1093/nop/npag024

**Published:** 2026-05-12

**Forthcoming Meetings**

**American Society of Clinical Oncology (ASCO) Annual Meeting**

May 29–June 2, 2026—Chicago, IL

https://www.asco.org/annual-meeting

**International Stereotactic Radiosurgery Society Congress**

May 31–June 3, 2026—Sydney, Australia

https://isrscongress.org/

**Asian Society for Neuro-Oncology (ASNO) Annual Meeting**

June 12–14, 2026—Kanazawa, Japan

https://site2.convention.co.jp/asno2026/

**22nd International Symposium on Pediatric Neuro-Oncology (ISPNO)**

June 28–July 1, 2026—Sydney, Australia

https://www.ispno2026.org/

**British Neuro-Oncology Society Annual Meeting (BNOS)**

July 1–3, 2026—Birmingham, UK

https://www.bnos.org.uk/bnos-2026/

**2026 SNO/ASCO CNS Metastasis Conference**

August 13–15—Boston, MA

https://2026snoasco.oa-event.com/

**SIOPE Brain Tumour Group Annual Meeting & Educational Course**

September 15–18, 2026—Athens, Greece

https://siope.eu/SIOPE-Brain-Tumour-Group

**European Association of Neuro-Oncology (EANO) Annual Meeting**

September 24–27, 2026—Rome, Italy

https://www.eano.eu/eano2026/

**Congress of Neurological Surgeons Annual Meeting**

October 31–November 4, 2026—Washington, D.C.

https://www.cns.org/annualmeeting

**Society of Neuro-Oncology Annual Meeting and Education Day**

November 12–15, 2026—Philadelphia, PA

https://sno2026.oa-event.com/

